# The Establishment of a Genetic Transformation System and the Acquisition of Transgenic Plants of Oriental Hybrid Lily (*Lilium* L.)

**DOI:** 10.3390/ijms24010782

**Published:** 2023-01-02

**Authors:** Yue Chen, Xinru Hou, Yupin Zheng, Yingmin Lyu

**Affiliations:** Beijing Key Laboratory of Ornamental Plants Germplasm Innovation & Molecular Breeding, China National Engineering Research Center for Floriculture, College of Landscape Architecture, Beijing Forestry University, Beijing 100083, China

**Keywords:** embryogenic callus, sterile scales, establishment of regeneration system, optimization of genetic transformation system, homologous transformation

## Abstract

Lily (*Lilium* spp.) has elegant flowers and beautiful colors, which makes it popular among people. However, the poor stress resistance and self-propagation ability of lily limit its application in landscaping to a great extent. In addition, transgenic technology is an important means to improve plant characteristics, but the lack of a stable and efficient genetic transformation system is still an important factor restricting the development of lily transgenic technology. Therefore, this study established a good lily regeneration system by screening different explants and plant growth regulators of different concentrations. Then, the genetic transformation system of lily was optimized by screening the critical concentration of antibiotics, the concentration of bacterial solution, and the infection time. Finally, the homologous lily cold resistance gene *LlNAC2* and bulblet generation gene *LaKNOX1* were successfully transferred to ‘Siberia’ and ‘Sorbonne’ to obtain lily transgenic lines. The results showed that when the stem axis was used as explant in ‘Siberia’, the induction rate was as high as 87%. The induction rate of ‘Sorbonne’ was as high as 91.7% when the filaments were used as explants. At the same time, in the optimized genetic transformation system, the transformation rate of ‘Siberia’ and ‘Sorbonne’ was up to 60%. In conclusion, this study provides the theoretical basis and technical support for improving the resistance and reproductive ability of Oriental lily and the molecular breeding of lily.

## 1. Introduction

Lily (*Lilium* spp.) belongs to the genus *Lilium* (Liliaceae) and is a perennial bulbous flower of the monocotyledonous subclass. It is one of the five world-famous fresh cut flowers and occupies an important share in the flower market [1]. Lily has an elegant appearance, rich colors, and high ornamental value [2]. It has important application value in cut flowers, potted plants, and landscaping. There are about 115 species of lilies [3], which can be grouped into seven types. Oriental lily is a common lily cultivar used for ornamental purposes. However, the cold resistance of Oriental hybrid lily is poor, and most of the Oriental lilies in horticultural production often use new bulbs to propagate every year. The high production cost limits their application in landscaping [4].

Transgenic technology is an important means to improve plant traits. Since the first transgenic plants were obtained in 1983, the research on plant genetic transformation has grown rapidly [5]. Genetic transformation is an important technical means to study gene function. Agrobacterium-mediated transformation is a commonly used method for plant transformation which employs an efficient and low-cost system and is widely used for stable gene transfer [6,7]. In 1992, Cohen first transformed lily bulbs by the Agrobacterium-mediated method and detected foreign genes in tumor-like protrusions [8]. Subsequently, with the development of molecular biotechnology, the application of genetic engineering technology in lily genetics and breeding has become more and more extensive, including *L. davidii Duchartre* [9], *L.× fomolongi* ‘Akasu’ [10], *L. longiflorum* [11], etc. Recently, genes related to plant resistance [12,13], flower development [14,15,16,17], bulb development [18], flower color [19,20], and flower fragrance [21,22] have been continuously developed, which provides good materials for the transgenic nature of lilies and provides a theoretical basis for further research. However, due to strong genotype dependence, poor genetic stability, and difficult regeneration of converted explants, this technology still cannot meet the requirements of genetic engineering for gene function verification and trait improvement [23]. Therefore, the establishment of an efficient lily regeneration system is also important to improve the conversion efficiency.

Lilies can be propagated in vitro using a variety of tissues and organs as explants, including cell suspensions, pollen, filaments, anthers, seeds, leaves, shoot tips, young stems, stem nodes, pseudo-bulblets, bulblets, and bulb scales, etc. In general, bulb scales are the most common explant type during lily bulb regeneration [24]. The plant growth regulators commonly used in plant tissue regeneration include Naphthylacetic acid (NAA), 2,4-Dichlorophenoxyacetic acid (2,4-D), Picloram (PIC), N6-benzyladenine (6-BA), Thidiazuron (TDZ), etc. Different plant growth regulators have different effects, and their different combinations will also exert different effects. For example, TDZ has dual activities of auxin and cytokinin and has been widely used in the induction of somatic embryos [25]. It is often used in combination with NAA or PIC. Therefore, it is necessary to screen plant growth regulators for different varieties to optimize the regeneration system.

In order to improve the stress resistance and self-reproduction ability of Oriental lily, homologous transformation was carried out to optimize the genetic transformation system through the study of gene function. The Longiflorum × Asiatic hybrid lily ‘Aladdin’ was found to have a very strong self-propagation ability under natural conditions, and it can spontaneously form a large number of underground stem bulblets of good quality on the underground stems [4]. It is an excellent test material for studying the formation mechanism of lily bulbs under natural conditions. Meanwhile, *Lilium lancifolium* Thunb., a wild species in northeast China, is mainly distributed in the northern temperate zone; it can survive at freezing temperatures and continues to germinate and bloom in the following spring. Therefore, it is an ideal research object to improve the cold tolerance of other plants [26,27]. In this study, through the early exploration by our research group, the LA lily ‘Aladdin’ bulblet gene *LaKNOX1* and the cold resistance gene *LlNAC2* were selected for homologous transformation.

The *KNOX1* gene selected in this study belongs to the homeobox gene family. Based on the sequence similarity, evolutionary relationship, and expression pattern, *KNOX* genes are divided into two subfamilies, namely, the class I *KNOX* subfamily and class II *KNOX* subfamily [28,29]. In the process of plant development, changes in the expression of class I *KNOX* family genes will lead to changes in cell fate, disruption of the differentiation process, and ultimately to the formation of new organs [30,31,32,33]. Class I *KNOX* genes are mainly expressed in shoot apical meristem (SAM) and immature vascular bundles, and they participate in maintaining the undifferentiated state of cells [34]. These genes were significantly inhibited before the initiation of lateral organ primordia, such as leaf primordia and flower primordia [35], indicating that their down-regulation can promote the breaking of the undifferentiated state of cells, which is of great significance for the formation of lateral organs [36]. In addition, overexpression of the *kn1* gene in maize directly causes ectopic meristem formation near leaf veins [30]. Similarly, ectopic lobule formation was also found on the edge of lettuce (*Lactuca sativa*) leaves overexpressing the *AtKNAT1/BP* gene [37]. This shows that the high expression of the class I *KNOX* gene can reverse the cell fate in the fully differentiated tissue to the uncertain state, thus leading to the formation of new meristem [30]. *KNOX* genes of class II are less studied [38]. Studies have shown that *KNOXII* is involved in the regulation of the secondary growth of plant cell walls and plays an important regulatory role in the development of roots, stems, seed coats, and heartwood [28,39,40,41,42,43,44,45]. In lilies, *LoATH1, LoSTM,* and *LoKNAT6* genes play a key role in bulblet formation, the initiation of secondary meristems, and the ability of explants to regenerate bulblets [46].

Another gene selected in this study, *LINAC2*, belongs to NAC transcription factor and plays an important role in regulating abiotic stress signal transduction in plants [47]. For example, under conditions such as drought, high salt, abscisic acid (ABA), methyl jasmonate (MeJA), and hydrogen peroxide (H_2_O_2_), the expression of the *Arabidopsis NAC* gene *ATAF1* can be induced, and the phenotype of overexpressed transgenic *Arabidopsis thaliana* includes dwarfism, shortened taproot, and enhanced salt tolerance and drought resistance [48]. In *Lilium Lancifolium*, the *LlNAC2* gene was shown to be significantly induced by low temperature, drought, salt stress, and abscisic acid treatment, while overexpression of the *LlNAC2* gene in tobacco showed stronger tolerance [49]. In addition, the regulation of abiotic stress by *NAC* genes in other plants has also been studied, such as *Cucurbita moschata CmNAC1* [50], *Suaeda glauca S1NAC8* [51], *Tamarix hispida ThNAC13* [52], and *Populus euphratica PeNAC036* [53], etc. They all enhanced the stress resistance of plants in different aspects.

The regeneration system was established by screening the explants and induction media of Oriental lilies ‘Siberia’ and ‘Sorbonne’. Then, the critical concentration of antibiotics, bacterial solution concentration, and infection time were screened to optimize the genetic transformation system. Finally, the ‘Aladdin’ bulblet gene *LaKNOX1* and the cold resistance gene *LlNAC2* of *Lilium lancifolium* Thunb. were homologously transformed to obtain transgenic lines, so as to improve the resistance and reproductive capacity of Oriental lily. It is of great significance to expand its application in landscaping, cultivate new varieties, and lay a foundation for its transgenic breeding.

## 2. Results

### 2.1. Effects of Different Plant Growth Regulator on Callus Induction of Two Kinds of Lily Stems

The stem axis explants of two varieties of oriental lilies were inoculated into six media with different plant growth regulators, in order to screen out the most suitable medium. It can be seen that the ‘Siberia’ stem (Table 1) has the highest induction rate, up to 80.7%, in N1 medium with NAA 1 mg/L + TDZ 0.1 mg/L, which is significantly different from other medium types, so that is the best medium for ‘Siberia’ callus induction. The induction effect of ‘Sorbonne’ lily (Table 2) was significantly worse than that of ‘Siberia’, and the induction rate was the highest in N1 medium with NAA 1 mg/L + TDZ 0.1 mg/L, but only 13.0%. There was no significant difference in the induction rate among the other five media except for N1 media. In summary, when the same explant is selected, the induction rate of different lily varieties may be significantly different.

**Figure 1:**
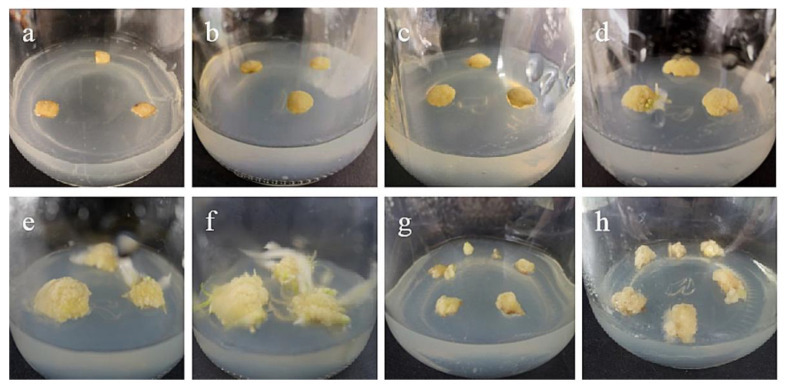
Callus induction from stem axis explants of ‘Siberia’: (**a**) 0 days after inoculation; (**b**) 15 days after inoculation, callus began to form; (**c**,**d**) callus increased gradually at 20, 30 days; (**e**,**f**) after 45 and 60 days of inoculation, a light yellow, compact callus with granular protuberances on the surface gradually formed; (**g**) the callus just subcultured; (**h**) the callus of subculture 20 days later.

Fifteen days after the inoculation of the stem axis, the incisions of ‘Siberia’ lily began to produce callus (Figure 1b), both in cross section and longitudinal section. Afterwards, the number of calli increased and gradually formed pale yellow, compact callus with granular protrusions on the surface (Figure 1e–f). In addition, ‘Sorbonne’ also induced callus, but the explants browned shortly after inoculation, and the explants took a long time to start, with only a very small number of calli induced in about 40–50 days. In conclusion, when the stem axis is an explant, the effect of ‘Siberia’ on callus induction is better than that of ‘Sorbonne’.

### 2.2. Effects of Different Plant Growth Regulators on Callus Induction of Two Kinds of Lily Filaments

The filaments of the two kinds of lilies were inoculated on the medium containing PIC and different concentrations of TDZ to screen out the best induction medium. It can be seen that the induction rate of ‘Siberia’ lily was significantly improved only in P2 medium supplemented with 0.1 mg/L TDZ, which was 47.2% (Table 3). In addition, the induction rate of ‘Siberia’ filaments was lower than that of ‘Sorbonne’, and it was very prone to browning, regardless of whether PIC was used alone or in combination with TDZ. Moreover, we can see that the induction rate of ‘Sorbonne’ lily in the P2 medium supplemented with 1 mg/L PIC + 0.1 mg/L TDZ was as high as 91.7% (Table 4). It can be concluded that P2 medium is the best medium for inducing callus of ‘Sorbonne’ filaments; and the filaments basically did not brown. The test results showed that the two Oriental lily cultivars both screened out the optimum hormone concentrations for filament-induced callus, but the highest filament induction rate of ‘Sorbonne’ was 44.5%, higher than that of ‘Siberia’. Therefore, ‘Sorbonne’ is more suitable for callus induction with filaments as explants. To sum up, even if the same explant is selected and the same growth regulator and its concentration are used, the induction rate and browning rate of different varieties are significantly different.

**Figure 2:**
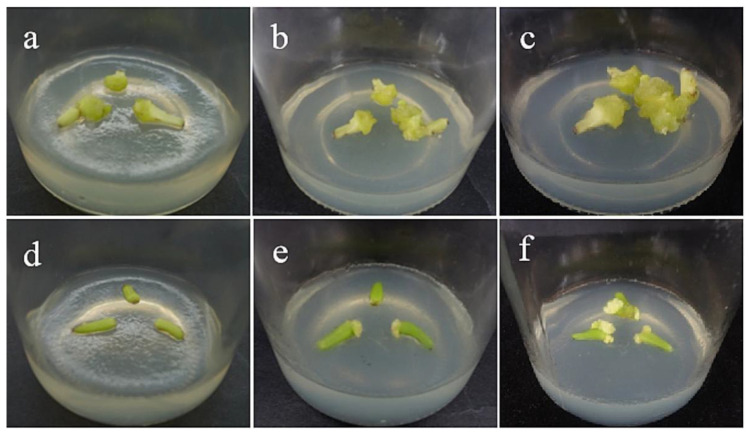
Callus induction of filament explants. (**a**–**c**) The bright yellow-green callus was obtained from the filaments of ‘Sorbonne’ at 35, 45, and 60 days. (**d**–**f**) The bright yellow-green callus was obtained from the filaments of ‘Siberia’ at 35, 45, and 60 days. The results showed that ‘Sorbonne’ began to produce callus on the 35th day, while Siberia began to produce callus on the 45th day, and the callus growth was slow. The callus volume induced by ‘Sorbonne’ filaments was significantly larger than that by ‘Siberia’.

After 15 days of inoculation, the morphological lower end of the filaments began to swell, and only individual explants began to form callus. After 30–35 days, the lower end of ‘Sorbonne’ continued to swell, with obvious callus production (Figure 2a), while ‘Siberia’ started to produce callus at about 45 days, and the callus grew slowly. However, the volume of callus induced by ‘Sorbonne’ filaments was significantly larger than that of ‘Siberia’, so from the comprehensive point of view of the induction rate and the amount of induction, ‘Sorbonne’ was more suitable for using filaments as explants to induce callus.

### 2.3. Morphological and Histological Observation of Callus

**Figure 3:**
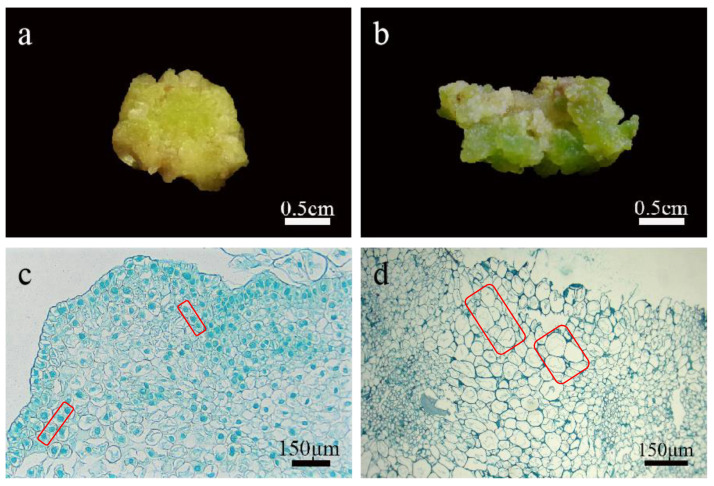
Identification of callus in lily. (**a**) The embryogenic calli were compact and granular; (**b**) the non-embryogenic calli were loose; (**c**) callus cells were small in volume, with large nuclei and easily stained, with few or no vacuoles, and the cell arrangement was relatively regular; (**d**) non embryogenic calli had a large volume, small nuclei, and were not easily stained, with large vacuoles and irregular cell arrangement.

**Figure 4:**
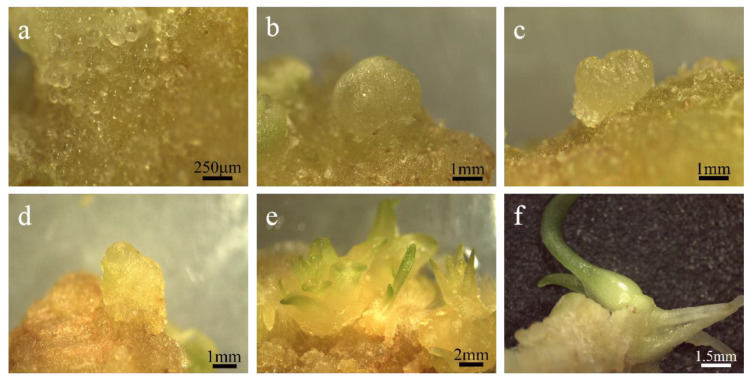
The regeneration of embryogenic callus. (**a**) Pre-embryo mass; (**b**) global embryo: the spherical protuberance seen on the surface of the explant is a spherical embryo; (**c**) heart-shaped embryo: the two sides of the top of the globular embryo grew rapidly and formed two cotyledon protrusions, making the embryo body heart-shaped; (**d**) torpedo shaped embryo: the cotyledon primordium of the heart-shaped embryo is further developed and elongated, and the cells in the cotyl region are elongated longitudinally while dividing, so that the shape of the embryo resembles the stage of a torpedo; (**e**) Cotyledon embryo: at this time, the cotyledons are slender and the embryo is close to maturity; (**f**) intact plantlets.

Callus can be divided into embryogenic callus and non-embryonic callus. Different explants and plant growth regulators induce different results. Even under the same conditions, callus with different shapes will appear, so the callus needs to be identified. The calli induced in this experiment were all compact and granular (Figure 3a), and under the microscope, numerous spherical preblastoids were seen protruding from their surface (Figure 4a), which were preliminarily identified as embryogenic calli. Then, through paraffin section observation, it was found that the callus cells induced in this experiment were small in size and the cell arrangement was relatively regular and strong. The cells had large nuclei and were easily stained, and they had few or no vacuoles (Figure 3c). However, non-embryogenic callus cells were large in size, with small nuclei and not easily stained. They had large vacuoles and irregular cell arrangements (Figure 3d). Based on the above observations, the calli induced in this experiment were identified as embryogenic calli.

Embryogenic callus will further develop into somatic embryos after regeneration and culture. Since somatic embryos are mostly originated from single cells, and the differentiation rates of different cells are different, we can observe somatic embryos at different stages on the same explant. In the process of embryogenic callus regeneration, it was observed by stereomicroscopy that it experienced four typical stages of embryonic development: spherical embryo, heart-shaped embryo, torpedo-shaped embryo, and cotyledon-shaped embryo (Figure 4b–e). When the cotyledon-shaped embryo grew to a certain stage, it further developed into a plantlet (Figure 4f). It is further proved that it is embryogenic callus through the development process of the callus. In the later experiments, it was found that the embryogenic callus induced by ‘Sorbonne’ filaments contained less embryogenic cells and formed less preblastoid than the embryogenic callus induced by the stem axis of ‘Siberia’. In addition, the embryogenic callus induced by ‘Sorbonne’ filaments will undergo the transition from embryogenic callus to non-embryogenic callus in the later subculture, which is called the loss of embryogenicity, and then these non-embryogenic calli will differentiate into distorted adventitious buds, which are difficult to differentiate. Comparing the two embryogenic calli, the callus induced by the ‘Siberia’ stem had better quality and state and was more suitable as a recipient material for genetic transformation.

### 2.4. Hygromycin Concentration Screening

**Figure 5:**
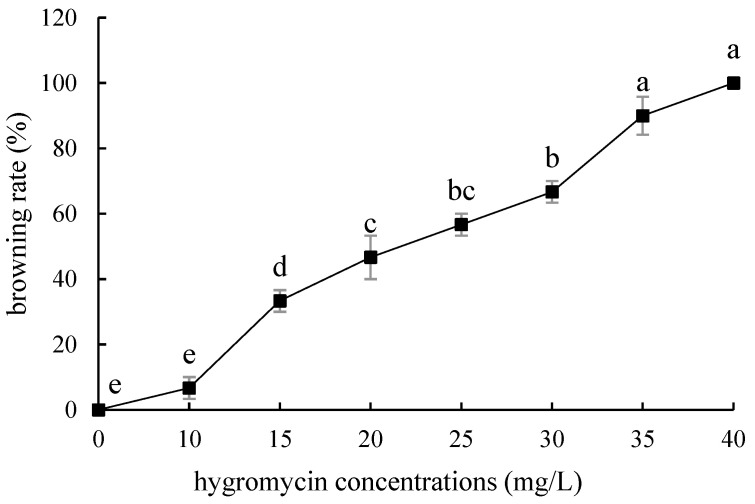
Browning rate of embryogenic callus of ‘Siberia’ under different hygromycin concentrations. Note: normal letters in every column indicate significant differences at 0.05 level by Duncan’s multiple range test.

**Figure 6:**
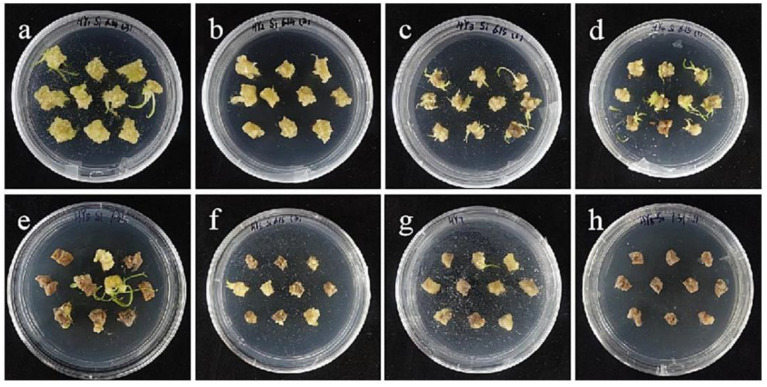
The selection process of hygromycin from embryogenic callus of ‘Siberia’. (**a**–**h**) The photos of embryogenic callus cultured on medium containing 0, 10, 15, 20, 25, 30, 35, and 40 mg/L hygromycin for 40 days. The results showed that at the concentration of 10–40 mg/L hygromycin, the browning rate was rising, and 40 mg/L hygromycin made all explants brown and die.

The embryogenic callus and small scales of sterile seedlings of two kinds of lilies were screened on the medium containing hygromycin. The results showed that the embryogenic callus of ‘Siberia’ was more sensitive to hygromycin. At the concentration of 10–40 mg/L hygromycin, the browning rate increased continuously (Figure 5); and at 35 mg/L, the browning rate of embryogenic callus was 90%, while 40 mg/L hygromycin caused all the explants to brown and die (Figure 6). In order to ensure the growth of transformed materials during the screening culture process, a hygromycin concentration slightly lower than the lethal concentration was generally selected, so 35 mg/L was used as the hygromycin screening concentration for ‘Siberia’ embryogenic callus.

**Figure 7:**
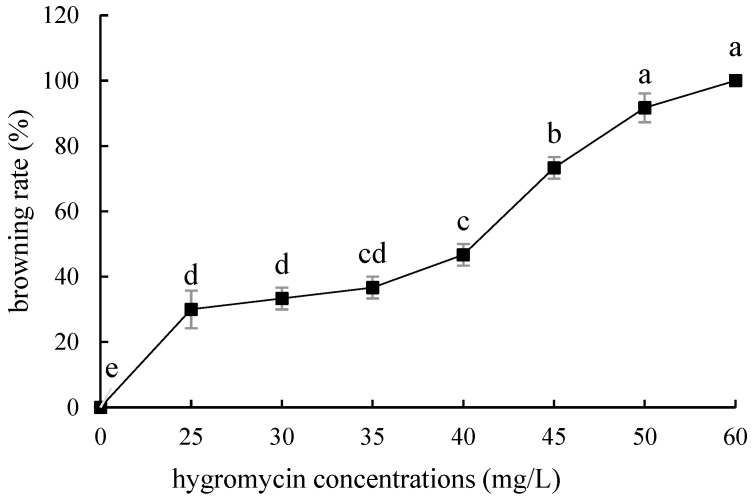
Browning rate of embryogenic callus of ‘Sorbonne’ under different hygromycin concentrations. Note: normal letters in every column indicate significant differences at 0.05 level by Duncan’s multiple range test.

**Figure 8:**
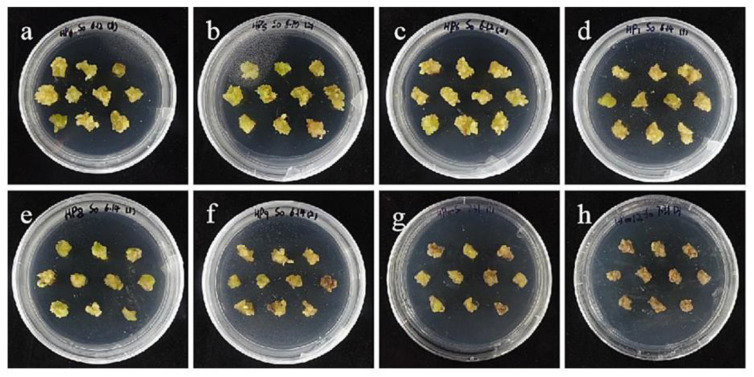
The selection process of hygromycin from embryogenic callus of ‘Sorbonne’. (**a**–**h**) Photos of embryogenic callus cultured on medium containing 0, 25, 30, 35, 40, 45, 50, and 60 mg/L hygromycin for 40 days. The results showed that when the concentration of hygromycin was 25–40 mg/L, there were different degrees of browning, but the browning was not serious. Generally, the part near the explant edge and the culture medium browned. When the concentration was 40–60 mg/L, the browning rate of ‘Sorbonne’ embryonic callus increased significantly, and the whole callus mass browned.

Compared with ‘Siberia’, ‘Sorbonne’ embryogenic callus is not very sensitive. When the concentration of hygromycin is 25–40 mg/L, there are different degrees of browning, but the browning is not serious (Figure 7). Generally, the explant edge and the part close to the medium were browned. When the concentration was 40–60 mg/L, the browning rate of the embryogenic callus of ‘Sorbonne’ increased significantly, and the whole callus was browned. Under the concentration of 50 mg/L, the callus lethality reached 91.7%, so 50 mg/L hygromycin was selected as the screening concentration of ‘Sorbonne’ embryogenic callus (Figure 8). It can be seen from Figure 5 and Figure 7 that different varieties of lily require different concentrations of screening antibiotics. Therefore, different varieties need to be screened separately.

**Figure 9:**
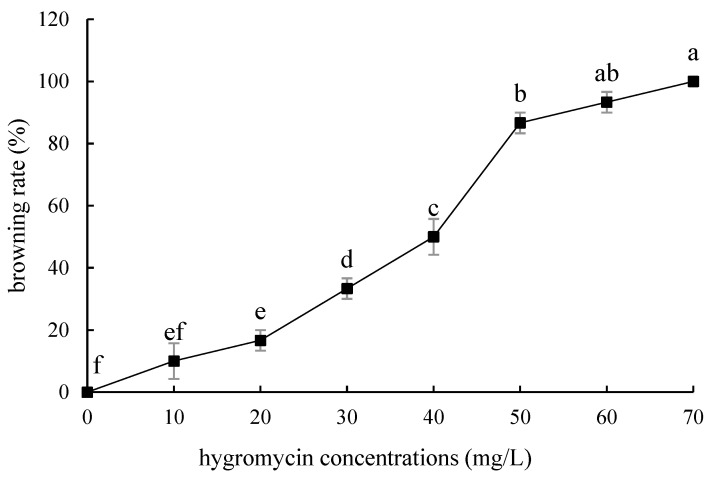
Browning rate of sterile scales of ‘Siberia’ under different hygromycin concentrations. Note: normal letters in every column indicate significant differences at 0.05 level by Duncan’s multiple range test.

**Figure 10:**
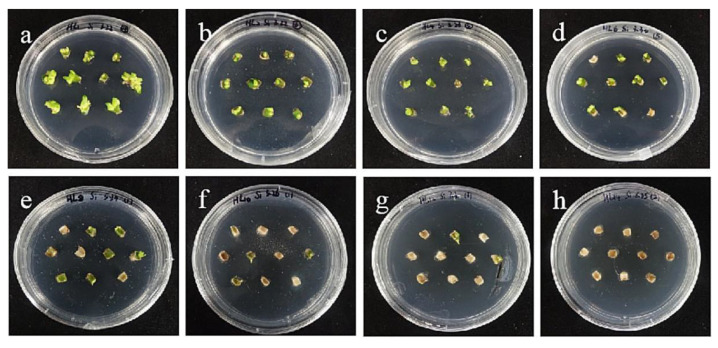
The selection process of hygromycin from sterile scales of ‘Siberia’. (**a**–**h**) The photos of embryogenic callus cultured on medium containing 0, 10, 20, 30, 40, 50, 60, and 70 mg/L hygromycin for 40 days. The results showed that in the range of 10–70 mg/L, with the increase in hygromycin concentration, the browning rate became higher and higher and the bud induction rate lower and lower. At 70 mg/L, all small scales were browning and died.

The tolerance of ‘Siberia’ sterile scales to hygromycin was higher than that of embryogenic callus, and with the increase in hygromycin concentration in the range of 10–70 mg/L, the browning rate became higher and higher, and the budding rate became lower and lower. At 70 mg/L, all the small scales turned brown and died (Figure 9), while the browning rate of the small scales reached 93.3% at 60 mg/L hygromycin concentration, and only a few small scales induced buds, so the hygromycin screening concentration of ‘Siberia’ small scales was 60 mg/L (Figure 10).

**Figure 11:**
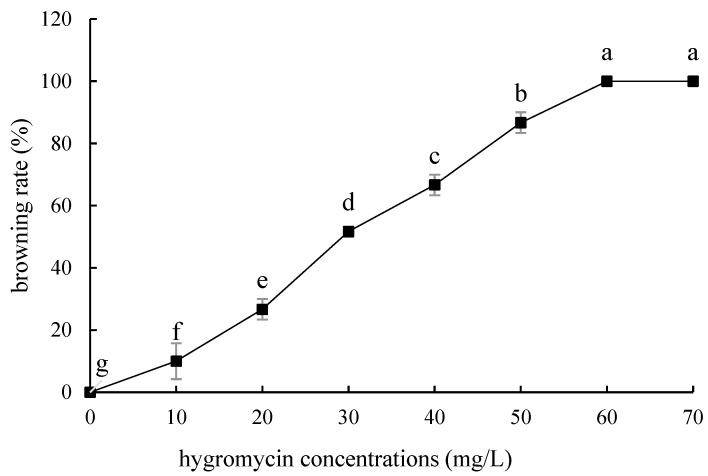
Browning rate of sterile scales of ‘Sorbonne’ under different hygromycin concentrations. Note: normal letters in every column indicate significant differences at 0.05 level by Duncan’s multiple range test.

**Figure 12:**
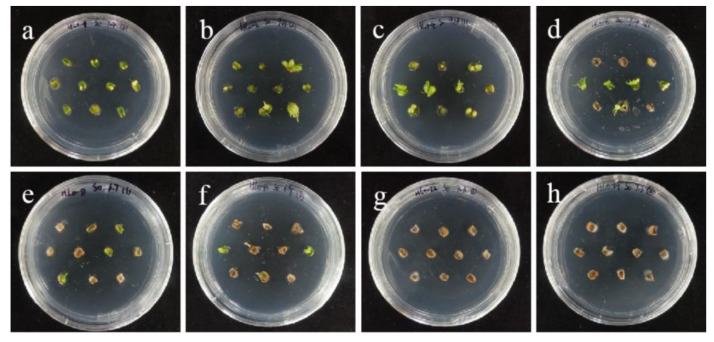
The selection process of hygromycin from sterile scales of ‘Sorbonne’. (**a**–**h**) The photos of embryogenic callus cultured on medium containing 0, 10, 20, 30, 40, 50, 60, and 70 mg/L hygromycin for 40 days. The results showed that with the increase in hygromycin concentration, small scales gradually lost their ability to induce buds, and all died at 60–70 mg/L.

The tolerance of ‘Sorbonne’ sterile scales to hygromycin was similar to that of embryogenic callus, but it was slightly more sensitive than that of ‘Siberia’. As the concentration of hygromycin increased, the small scales gradually lost their ability to induce buds (Figure 11). The browning rate of 50 mg/L hygromycin reached 86.7%, and all died at 60–70 mg/L (Figure 12). The screening concentration of hygromycin for the scales was 50 mg/L. Therefore, the screening concentration of hygromycin for ‘Sorbonne’ small scales was 50 mg/L.

### 2.5. Cephalosporin Concentration Screening

**Figure 13:**
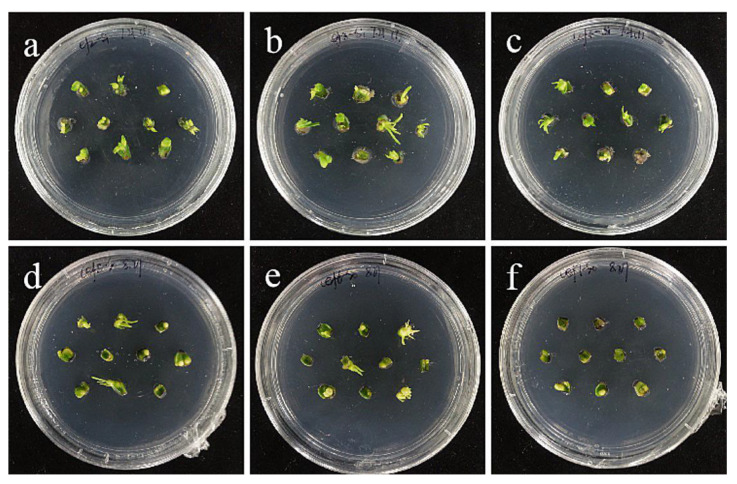
The selection process of cefotaxime from sterile scales. (**a**–**c**) The photos of ‘Siberia’ sterile scales after 30 days of screening on cefotaxime medium of 100, 200, and 250 mg/L; the results showed that in the low-concentration antibiotic medium of 100–200 mg/L, there were sporadic spots of contamination, and there was no growth of bacteria in the high concentration of bacteriostatic. (**d**–**f**) The photos of ‘Sorbonne’ sterile scales after 30 days of screening on cefotaxime medium of 300, 350, and 400 mg/L.

Cephalosporin is less harmful to plant cells and is commonly used as bacteriostatic antibiotics in Agrobacterium-mediated plant genetic transformation. In this experiment, the embryogenic callus and the small scales of sterile seedlings of two Oriental lilies were screened for the concentration of cephalosporin. It can be seen that after the small scales of the sterile seedlings were infected by Agrobacterium, the growth of bacteria was not obvious (Figure 13). Considering both the pollution situation and the germination rate, the intermediate concentration of 300 mg/L is the concentration of bacteriostat in the later transformation and screening process of Oriental lily.

**Figure 14:**
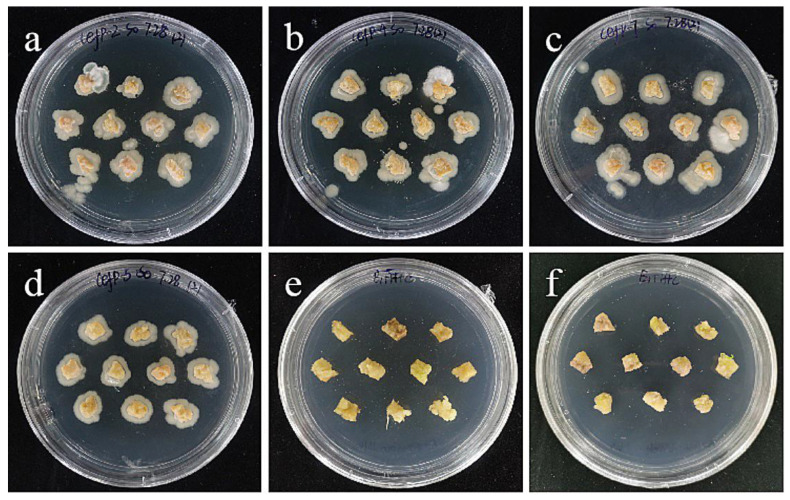
The selection process of cefotaxime from sterile scales. (**a**–**c**) The photos of embryogenic callus cultured on cefotaxime medium of 100, 250, and 400 mg/L for 30 days; the results showed that the embryogenic callus had a very serious bacterial overflow on the 0–400 mg/L cephalosporin medium, and a large number of Agrobacterium colonies grew on the surface and around the explants. (**d**–**f**) The photos of embryogenic callus cultured on cephalosporin medium containing 300 mg/L for 30 days after infection at the concentration of bacterial solution of 0.6, 0.4, and 0.2; the results showed that after infection with a low bacterial concentration of 0.2–0.4, there was no bacterial overflow when inoculated on the medium containing 300 mg/L cephalosporin.

However, embryogenic callus has a very serious phenomenon of excessive bacteria on 0–400 mg/L cephalosporin medium, and a large number of Agrobacterium colonies grow on the surface and surrounding the explants (Figure 14). Even 400 mg/L cephalosporin had no antibacterial effect, which may be due to the strong granularity on the surface of embryogenic callus and the adhesion and residue of Agrobacterium, so it led to the excessive growth of Agrobacterium. Moreover, the higher the concentration of bacteria liquid, the more Agrobacterium adheres and remains. Therefore, a low bacterial concentration of 0.2–0.4 was used to inoculate it on the medium containing 300 mg/L cephalosporin, and it was found that there was no overflow phenomenon (Figure 14), and almost no colonies appeared, indicating that the concentration of bacterial liquid may be the precondition for screening the concentration of embryogenic bacteriostat. Even a high concentration of bacteriostat cannot solve the excessive bacterial growth caused by high bacterial concentration. Combined with the screening results of the small scales, 300 mg/L was used as the concentration of embryogenic bacteriostat.

### 2.6. Effects of Bacterial Concentration and Infection Time on Embryogenic Callus

**Figure 15:**
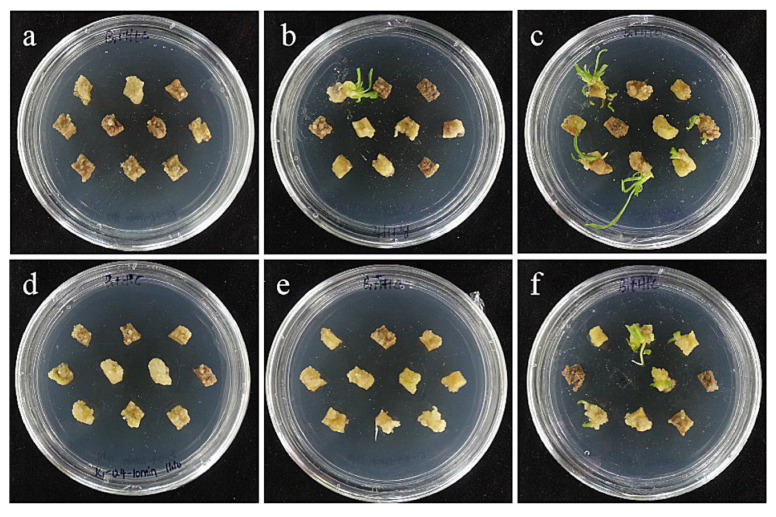
The photos of embryogenic callus of ‘Siberia’ screened for 60 days under different transformation conditions. (**a**) OD600 = 0.2, 10 min; (**b**) OD600 = 0.2, 15 min; (**c**) OD600 = 0.2, 20 min; the results showed that when the concentration of bacterial solution was 0.2, the transformation rate of ‘Siberia’ embryogenic callus increased with the increase in infection time; (**d**) OD600 = 0.4, 10 min; (**e**) OD600 = 0.4, 15 min; (**f**) OD600 = 0.4, 20 min; the results showed that when the concentration of bacterial solution was 0.4, the transformation rate first increased and then decreased with the increase in infection time.

The combined effect of the concentration of the infection solution and the infection time has a certain influence on the genetic transformation efficiency. The results showed that the Agrobacterium concentration of 0.4 and the infection time of 15 min were the optimal transformation conditions for embryogenic callus of ‘Siberia’ (Table 5; Figure 15).

### 2.7. Effects of Bacterial Concentration and Infection Time on Small Scales of Sterile Seedlings

**Figure 16:**
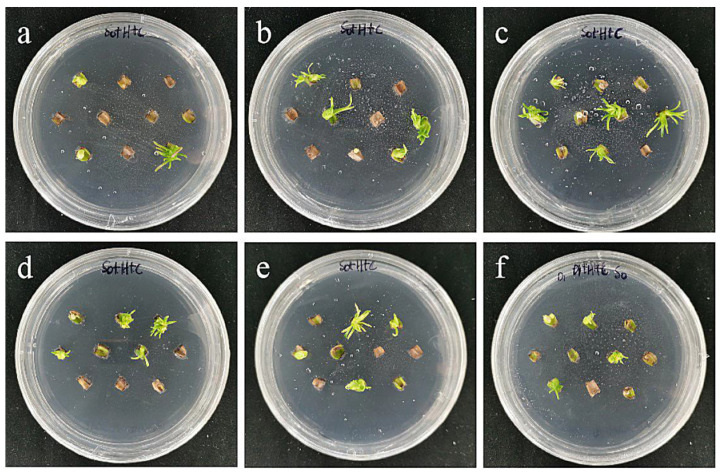
The photos of sterile scales of ‘Sorbonne’ screened for 60 days under different transformation conditions. (**a**) OD600 = 0.6, 10 min; (**b**) OD600 = 0.6, 15 min; (**c**) OD600 = 0.6, 20 min; the results showed that the transformation rate of ‘Sorbonne’ small scales increased with the increase in infection time at the concentration of 0.6 bacterial solution; (**d**) OD600 = 0.8, 10 min; (**e**) OD600 = 0.8, 15 min; (**f**) OD600 = 0.8, 20 min; the results showed that when the concentration of bacterial solution was 0.8, the transformation rate had a downward trend with the extension of the infection time.

The results showed that the optimal transformation conditions for the small scales of the sterile seedlings of ‘Sorbonne’ were an Agrobacterium OD600 value of 0.6 and infection time of 20 min (Table 6; Figure 16).

**Figure 17:**
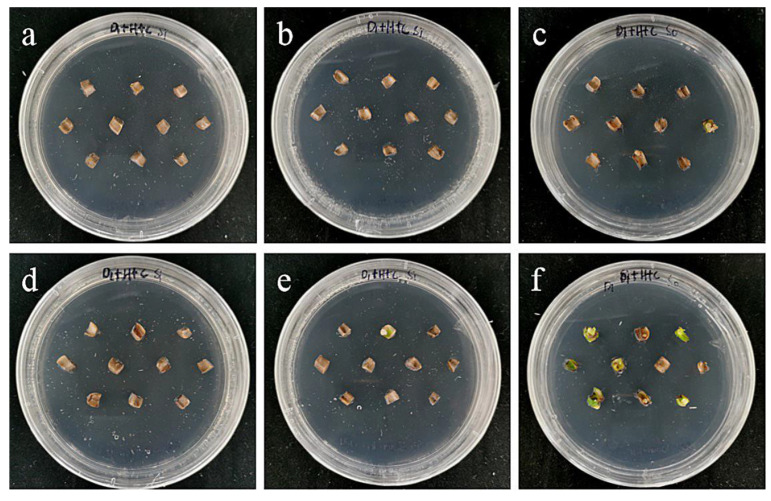
The photos of sterile scales of ‘Siberia’ screened for 60 days under different transformation conditions. (**a**) OD600 = 0.6, 10 min; (**b**) OD600 = 0.6, 15 min; (**c**) OD600 = 0.6, 20 min; (**d**) OD600 = 0.8, 10 min; (**e**) OD600 = 0.8, 15 min; (**f**) OD600 = 0.8, 20 min; the results showed that the transformation rate of the small scales of ‘Siberia’ sterile seedlings under six infection conditions was not ideal, and only a slight transformation rate was obtained when the concentration of bacterial solution was 0.8 and the infection time was 20 min.

The transformation rate of the ‘Siberia’ sterile seedling scales under the six infection conditions was not ideal; only the transformation rate of 13.3% was achieved under the conditions of the bacterial concentration of 0.8 and the infection time of 20 min (Table 7; Figure 17). There were basically no resistant buds, but this also shows a trend. The ‘Siberia’ small scales may require a higher bacterial concentration and time for infection to be conducive to genetic transformation. Therefore, 0.8 or more can be considered in subsequent studies. The concentration of bacterial solution was optimized for transformation conditions.

### 2.8. Transformed Plant Detection

**Figure 18:**
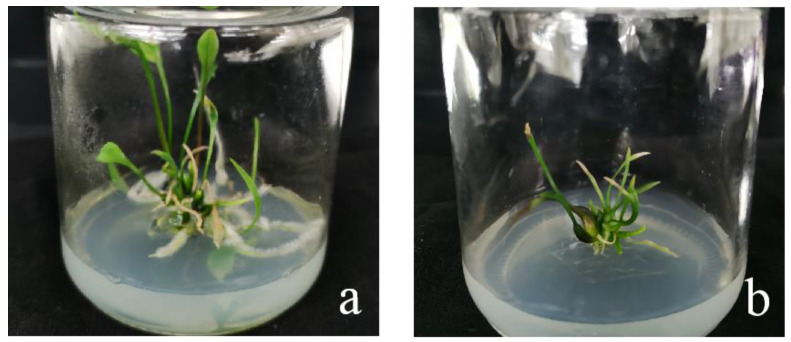
‘Siberia’ and ‘Sorbonne’ resistant plants. After 2 months of screening and 2–3 months of regeneration culture in Siberia and Sorbonne infected by Agrobacterium, the resistant plants were obtained. (**a**) ‘Siberia’; (**b**) ‘Sorbonne’.

When the recipient material was infected with Agrobacterium, after 2 months of screening and 2–3 months of regeneration, ‘Siberia’ obtained 31 resistant plants through the somatic embryogenesis pathway of the embryogenic callus, and ‘Sorbonne’ obtained 49 resistant plants from sterile seedling scales (Figure 18).

**Figure 19:**
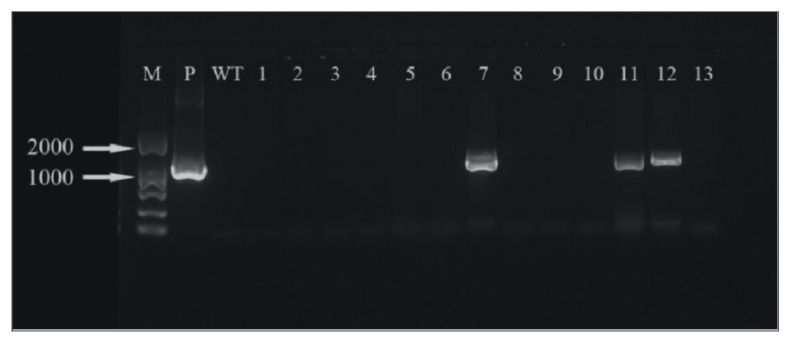
‘Siberia’ PCR test results. M: marker; P: positive control; WT: non-transformed plants; 1–13: transformed plantlets.

**Figure 20:**
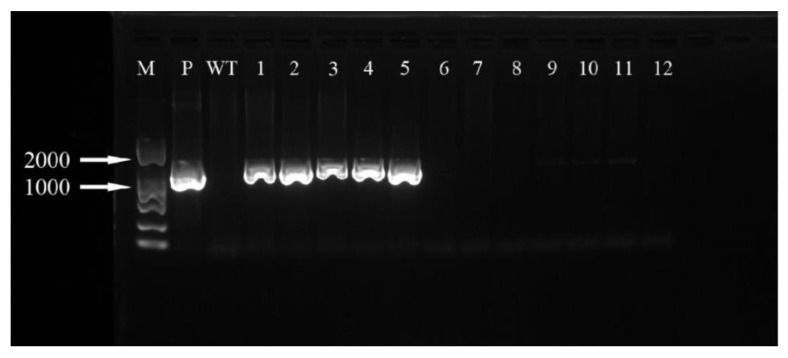
‘Sorbonne’ PCR test results. M: marker; P: positive control; WT: non-transformed plants; 1–12: transformed plantlets.

The hygromycin resistance gene was amplified by PCR and analyzed by agarose gel electrophoresis. It can be seen that three (Figure 19) and five (Figure 20) of the ‘Siberia’ and ‘Sorbonne’ resistant plants have amplified specificity at 1026 bp (the length of the hygromycin resistance gene), respectively. The size of the band is consistent with that of the positive control plasmid. In addition, the transformed plants and the remaining resistant seedlings have no specific bands. It is preliminarily shown that the foreign T-DNA gene has been integrated into the genomes of ‘Siberia’ and ‘Sorbonne’ lily.

## 3. Discussion

In this study, oriental hybrid lilies ‘Siberia’ and ‘Sorbonne’ were selected as the research objects to establish a transformation receptor system, optimize the genetic transformation system, and improve their cold resistance and reproductive ability through genetic transformation.

### 3.1. Effect of Explants on Embryogenic Callus Induction

There are many kinds of explants that can induce embryogenic callus in lily, including cell suspensions, pollen, filaments, anthers, seeds, leaves, shoot tips, young stems, stem nodes, pseudo-bulblets, bulblets, and bulb scales, etc. [24]. Moreover, genotype is an important factor affecting regeneration ability [54]. However, there are many genotypes of lily, and different genotypes have significant differences in their ability to induce embryogenic callus. Sun et al. used the filaments and ovaries of six wild lily species as explants to induce embryogenic callus and found that *Lilium regale* Wilson. had the best induction ability when using filaments, while *Lilium brownii* had the highest induction rate when using ovaries [55]. In this study, stem axis and filament were selected as explants. The results showed that the explants suitable for the callus induction of two lily varieties were different. It was further confirmed that even if the same explants were selected, different lily varieties also had different induction rates. Therefore, we can select different organs and materials to obtain higher induction rates and improve the regeneration system of lilies.

### 3.2. Effect of Plant Growth Regulators on Embryogenic Callus Induction

The types and concentrations of plant growth regulators are important factors affecting the induction rate. Thiazine (TDZ) has dual activities of auxin and cytokinin and has been widely used in the induction of somatic embryos [25]. It is often used in combination with auxin naphthyl acetic acid (NAA) or picloram (PIC). When the stem axis is the explant, it is suitable for the hormone combination of NAA and TDZ [56]. This experiment found that when NAA (1 mg/L) and TDZ were used together, the induction rate and the ratio of NAA to TDZ had a certain relationship, and different varieties were suitable for different hormone concentrations.

PIC has a significant effect on plant somatic embryo induction, embryogenic maintenance, and somatic embryogenesis, and it is often used for flower organ induction [57]. Only adding PIC can induce embryogenic callus, but the induction rate is relatively low. Some studies have combined PIC with TDZ or NAA to induce lily scales, and the induction rate is ideal [58], which is better than the induction result of only adding PIC. In this experiment, our study further confirmed that the induction rate of the PIC + TDZ combination was generally higher than that of PIC alone. Therefore, we also need to screen plant growth regulators for different lily varieties.

### 3.3. Regeneration of Embryogenic Callus

Somatic embryogenesis can be divided into two pathways, direct and indirect. Direct somatic embryogenesis refers to the direct differentiation of ex vivo explants into embryoid bodies by tissue culture using a suitable medium. Indirect somatic embryogenesis means that the explants in vitro are first dedifferentiated on the induction medium to form embryogenic callus, and then differentiated into embryoid bodies [59]. The embryogenic callus will go through several stages similar to globular embryo, heart-shaped embryo, torpedo-shaped embryo, cotyledon-shaped embryo, and zygotic embryo [60]. In this study, during the regeneration process, we also observed the globular embryo, heart-shaped embryo, torpedo-shaped embryo, and cotyledon-shaped embryo, which were similar to zygotic embryogenesis. However, the embryogenic callus induced by ‘Sorbonne’ filament is not easy to differentiate, which may be due to the fact that there are fewer embryogenic cells on the surface of the callus, the embryogenic state of the callus remains unstable, and the transition from embryonic callus to non-embryonic callus occurs in the later succession process, resulting in the loss of embryonic callus. It may also be that the differentiation of ‘Sorbonne’ embryogenic callus requires the addition of specific plant growth regulators, and the basic medium cannot meet its differentiation needs. Therefore, in the subsequent experiment, we can consider screening and optimizing the culture medium for the maintenance and proliferation of ‘Sorbonne’ embryonic callus. This means that the genetic transformation system is affected by many factors. We need to optimize different conditions to maximize the transformation efficiency.

### 3.4. Antibiotic Concentration Screening and Bacteriostatic Concentration Screening

In the process of transgenic breeding, the appropriate concentration of labeled antibiotics can effectively inhibit the growth of non-transformed recipients but does not affect the normal growth of transformed plants, which is a key step for the success of genetic transformation. In the research of lily genetic transformation, hygromycin, kanamycin, and glyphosate are commonly used as resistance screening agents [61,62]. This study found that the appropriate antibiotic concentration of ‘Siberia’ is quite different from that of ‘Sorbonne’. It can be seen that when the explant material is the same, the appropriate antibiotic concentration of different lily varieties is different, and even if it is the same variety, the antibiotic concentration corresponding to different receptor types is also different. Both the previous studies and the results of this experiment indicate that it is necessary to screen the concentration of antibiotics for different varieties and receptor materials.

Another type of antibiotic used in genetic transformation is bacteriostatic antibiotic. Antibacterial antibiotics are mainly used to prevent the receptor from being polluted after being infected by Agrobacterium and to reduce or avoid the death of transformation materials and regeneration difficulties caused by inhibiting the growth of Agrobacterium. Cephalosporin has wide resistance; it can effectively inhibit the growth of Agrobacterium, and it is less harmful to plant cells. It is a commonly used bacteriostatic agent in the process of plant genetic transformation [63]. At the same time, this study found that the concentration of the bacteriostatic agent was related to the concentration of Agrobacterium. When the low concentration of bacterial solution was used to infect, almost no colonies appeared. It is speculated that the concentration of Agrobacterium was the prerequisite for obtaining the screening results of the bacteriostatic agent. Therefore, it is necessary to combine the concentration of the bacterial solution and the bacteriostatic concentration for screening to reduce the pollution of receptor materials.

### 3.5. Effects of Bacterial Concentration and Infection Time on Genetic Transformation

The concentration of Agrobacterium and the infection time have important effects on the transformation efficiency. If the concentration of the bacterial solution is too low or the infection time is too short, the Agrobacterium cannot fully adhere to the recipient tissue and thus cannot achieve effective transformation. If the concentration of the bacterial solution is too high or the infection time is too long, it will cause serious damage to the recipient material [23,64]. In this study, it was confirmed that there was little bacterial spillage at a low bacterial concentration. In addition, we also found that the embryogenic callus was more sensitive to the external environment than the small scale. In this experiment, the suitable concentration of bacterial solution or infection time of sterile seedling scales was higher than that of embryogenic callus. That is, different explants are suitable for different transformation conditions. At the same time, with the same sterile seedling scale as the explant, the screening results obtained by ‘Siberia’ and ‘Sorbonne’ are quite different, which shows that even with the same explant, the best transformation conditions of different varieties are different.

### 3.6. Positive Detection of Transformed Plant

Most of the resistant seedlings obtained in this experiment were found to be false positive plants without amplification of target bands by PCR detection. Compared with model plants such as *Arabidopsis* and tobacco, lily is more likely to produce pseudo-transformants, presumably due to its complex genotype. At the differentiation stage after screening, the resistance screening of the regeneration medium was not carried out, which may have resulted in the growth of non-transformed cells due to the differentiation process of embryonic callus and the rooting process of small-scale adventitious buds, and the false positive rate was increased. Therefore, to improve the positive rate of the transformed plants, we need to pay attention to the use of receptors with a more uniform state and screen the concentration of antibiotics in the regeneration stage. If the acquired resistant seedlings are kept in hygromycin medium, the false positive rate will be effectively reduced.

The receptor system and genetic transformation system are the key contents of lily molecular breeding. In this study, the author established a good receptor system and optimized the genetic transformation system for Oriental lily. The obtained resistant plants have been identified and initially determined to be transgenic plants. In the follow-up study, GUS histochemical analysis will be used to further identify whether the foreign gene has been successfully introduced. In addition, after the plants have grown, phenotypic identification of the transgenic plants and studies on bulb formation and cold stress will be carried out to verify the functions of the target genes *LaKNOX1* and *LlNAC2*, thereby improving the shortcomings of poor cold resistance and difficult reproduction of Oriental lily.

## 4. Materials and Methods

### 4.1. Plant Materials, Agrobacterium Strain, and Plasmid

The experimental materials were stems and filaments of Oriental lilies ‘Siberia’ and ‘Sorbonne’. The bulbs were purchased from Haining Huahai Horticultural Co., Ltd. (Haining, China), and some bulbs were planted in the Beijing Forestry University Science and Technology Greenhouse and used to pick flower buds two months later. For media recipes, refer to Table A1. In this study, *Agrobacterium tumefaciens* GV3101, pBI121-*LaKNOX1*-GFP, and pBI121-*LlNAC2*-GFP plasmids constructed in the early stage of the experiment; plant expression vector pCAMBIA1300-GUS; and *A. tumefaciens* strain GV3101 were used.

### 4.2. Induction of Embryogenic Callus

Select the bulbs of Oriental hybrid lilies ‘Siberia’ and ‘Sorbonne’, peel off the scales to obtain the central stem and soak it in detergent water for 15 min, and then rinse with running water for 4 h. Then, place it on an ultra-clean workbench, then disinfect with 75% alcohol for 30 s, then rinse with sterile water 2–3 times, then disinfect with 3% sodium hypochlorite for 15 min and rinse with sterile water 5–6 times, and finally place it in an inoculation tray with sterile paper to drain. After draining, cut the stems transversely into 0.1 cm thin slices as explants.

Take 5–6 cm flower buds of Oriental hybrid lilies ‘Siberia’ and ‘Sorbonne’, soak in detergent water for 15 min, then rinse with running water for 2 h. Then, place it on the ultra-clean workbench, then disinfect with 75% alcohol for 30 s, then rinse with sterile water for 2–3 times, then disinfect with 1.5% sodium hypochlorite for 15 min and rinse with sterile water for 5–6 times, and finally place it in an inoculation tray with sterile paper to drain. After draining, cut the buds, take out the filaments, and cut them to about 5 mm to be used as explants.

Inoculate the stem and filament explants of the two lilies into different primary media (Table A1). Replicate each of the above treatments 3 times with 18 explants per replicate and subculture every 30 days. Culture the cells in the dark at 25 ± 2 °C and count the induction rate of primary callus after 60 days.

### 4.3. Histological Identification of Callus

Cut the induced callus to 5 × 5 mm in size and 3 mm in thickness and photograph under a stereomicroscope. Put the sample into FAA fixative solution for fixation (more than 24 h). The amount of fixative is 20 times that of the material [65]. In order to infiltrate the fixing liquid into the material as soon as possible, a vacuum pump is used to evacuate the fixing liquid. Then, use 30%, 50%, 70%, 85%, 95%, and 100% concentrations of alcohol for 30–60 min dehydration, then transfer to xylene: anhydrous alcohol = 1:1, pure xylene (twice) transparent at all levels, for 40 min; add the material to 25%, 50%, and 75% paraffin–xylene solution for dipping in wax and 30 min in the incubator for each stage, and then change the pure wax 3 times. When embedding, place the material according to the position to be observed; trim the embedded wax block, slice it, cut it into continuous wax strips, and put it in warm water at 45 °C to spread it. Stick it with plum protein patch. Apply a thin layer of plum protein patch on the slide, place it on the slide for a while, then drop distilled water on the slide with a dropper, and then take a section of wax tape and place it on the water surface of the slide. Place the smooth side of the paraffin tape on the water, and then place the slide on the temperature stage to slowly heat it, so that the wax tape can expand freely on the water. Paraffin sections were performed with reference to Zhou et al. (2018) [66].

Dry the pasted sections for one to several days, and then dewax, rehydrate, and stain. Use safranin and fast green staining. Paraffin section → dewaxing in xylene (5 min) → 1/2 xylene + 1/2 pure alcohol (2 min) → 100% alcohol (2 min) → 95% alcohol (2 min) → 85% alcohol (2 min) → 70% alcohol (2 min) → 50% alcohol (2 min) → distilled water (2 min) → 1% safranine dye (30 min) → distilled water → 50% alcohol (2 min) → 70% alcohol (2 min) → 85% alcohol (2 min) → 95% alcohol (2 min) → 0.1% or 0.5% solid green dye (10–40 s) → 95% alcohol → 100% alcohol (2 min) → 1/2 xylene + 1/2 pure alcohol → xylene transparent (5 min) → gum seal. Observe the prepared paraffin sections under a microscope and photograph [67].

### 4.4. Regeneration of Embryogenic Callus

For media recipes, refer to Table A1. The culture environment was 25 ± 2 °C and the light was 8 h/16 h.

### 4.5. Scale-Induced Plant Regeneration

Strip the middle and outer scales of the bulbs of Oriental lilies ‘Siberia’ and ‘Sorbonne’, and cut off the diseased part. The pretreatment and disinfection methods were the same as the stem axis. Cut into squares of about 0.5 cm^2^ on the ultra-clean workbench as explants and inoculate them into different primary media (Table A1). The culture environment was 25 ± 2 °C and the light was 8 h/16 h. The obtained adventitious buds grow to 2 cm and then add them to the rooting culture and bulb expansion culture media (Table A1).

### 4.6. Plant Overexpression Vector Construction

According to the full length of *LaKNOX1*, the *LlNAC2* gene ORF (stop codon removed) and the restriction site sequence on the pCAMBIA1300-GUS expression vector, *XbaI* (TCTAGA) and *SalI* (GTCGAC), were selected for double restriction digestion, and then we designed specific primers with restriction sites for transgenic lily according to the instructions of Vazyme’s ClonExpress^®^ II One Step Ligation Kit (Table A2).

We used the pBI121-*LaKNOX1*-GFP and pBI121-*LlNAC2*-GFP plasmids stored in the laboratory as templates, and then PCR technology was used to amplify the target gene fragments containing restriction sites. After detection by 1% agarose gel electrophoresis, the correct band was recovered using the AxyPrepTM DNA gel recovery kit (Table A3).

The pCAMBIA1300-GUS vector plasmid was double digested with *Xbal* and *SalI*. After 6 h of digestion at 37 °C, we used gel electrophoresis to check whether it was cut (the circular plasmid without cutting runs faster than the cut linear plasmid). Finally, the AxyPrepTM PCR cleaning kit was used to purify and recover the double enzyme digestion product, and the concentration was measured for later use (Table A4).

We used the ClonExpress^®^ II One Step ligation kit to ligate the target gene fragment containing the restriction site with the linear vector, and it was incubated at 37 °C for 30 min (Table A5). The ligation product was transformed into *E. coli* DH5α competent cells; the transformed product was spread on LB medium containing Kan and cultured at 37 °C overnight, and the monoclonal colonies were identified by PCR. Finally, the bacteria with correct PCR results were selected and sent to a company for sequencing. Sequencing was performed by Beijing Ruibo Xingke Biotechnology Co., Ltd. The recombinant plasmids with correct sequencing results were transformed into *A. tumefaciens* strain GV3101.

### 4.7. Screening of Critical Concentrations of Hygromycin and Cephalosporin

Hyg concentration gradient was set to 0, 10, 15, 20, 25, 30, 35, 40 mg/L, then added to the ‘Siberia’ embryogenic callus optimal induction medium. The Hyg concentration gradient was set to 0, 25, 30, 35, 40, 45, 50, 60 mg/L, then added to the optimal induction medium of ‘Sorbonne’ embryogenic callus, and each culture dish was inoculated with 10 0.5 cm^3^ calli. The treatment was repeated 3–6 times and cultured in the dark at 25 ± 2 °C, and the browning was counted after 60 days.

Hyg concentration gradient was set to 0, 10, 20, 30, 40, 50, 60, 70 mg/L, then added to ‘Siberia’ and ‘Sorbonne’ scale induction media, inoculated with 100.5 cm^2^ per dish of small scales of sterile seedlings. Each treatment was repeated 3–6 times and incubated at 25 ± 2 °C, 8 h/16 h light, and the browning was counted after 60 days.

Cef concentration gradients were set at 0, 100, 200, 250, 300, 350, and 400 mg/L. After Agrobacterium infection, embryogenic callus of ‘Siberia’ and ‘Sorbonne’ and small scales of sterile seedlings were inoculated into the induction media containing different concentrations of Cef (Table A1). Each culture dish was inoculated with 10 receptors, each treatment was repeated 3–6 times, and the contamination of the receptor material was observed after 30 days.

### 4.8. Preparation of Agrobacterium

The pCAMBIA1300-GUS recombinant plasmid was transformed into *A. tumefaciens* strain GV3101, and the positive transformants were screened with antibiotics (50 mg/L Rif and 50 mg/L Kan) and identified by PCR. The correctly identified transformants were inoculated in LB liquid medium containing 50 mg/L Kan and 50 mg/L Rif, and cultured in a shaker at 28 °C, 200 r/min for 16–18 h. Then, we transferred the bacterial solution to 50 mL at the ratio of 1/20 LB liquid medium (containing 50 mg/L Kan + Rif), 28 °C, 200 r/min shaking culture for 16–18 h. We centrifuged the tube at 4 °C and 5000 r/min for 10 min, discarded the supernatant, then resuspended the bacteria with the infection solution, and adjusted the concentration of the bacterial solution until the OD600 was 0.2, 0.4, 0.6, 0.8 for later use.

### 4.9. Receptor Material Pre-Incubation

The embryogenic calli of ‘Siberia’ and ‘Sorbonne’ were placed in the optimal induction medium, and the small scales of sterile seedlings were placed in the scale induction media (Table A1) and pre-cultured for 3 days at 25 ± 2 °C in the dark.

### 4.10. Infection and Co-Culture

We infected the transformed recipient with the infection solution prepared in Section 4.7; the OD600 of the embryogenic callus was 0.2, 0.4; the OD600 of the small scales of sterile seedlings was 0.6, 0.8; and the infection time was set to 10, 15, 20 min. There were 6 different infection conditions designed for each receptor material. After infection, the cells were transferred to a co-culture medium and cultured in the dark for 3d (Table A1).

### 4.11. Screening Culture

The recipient material after co-cultivation was transferred to the screening medium (Table A1). Embryogenic callus was cultured in the dark, and the small scales of sterile seedlings were cultured in the light for 8 h/16 h, and they were screened at 25 ± 2°C for 60 days. Finally, the callus survival rate and the germination rate of small scales were calculated.

### 4.12. Cultivation of Resistant Seedlings

The resistant calli and adventitious buds obtained in Section 4.11 were inoculated into MS medium and cultured for about 2 months until resistant seedlings were obtained, which were used for the later detection of transgenic plants.

### 4.13. PCR Detection of Transformed Plants

According to the DNA extraction kit (DN14) of Beijing Aidelai Biological Reagent Co., Ltd. (Beijing, China), the genomic DNA of the tissue culture seedlings of the obtained hygromycin-resistant plants was extracted according to the instructions. Using plasmid pCAMBIA1300-GUS, DNA of non-transgenic plants, and DNA of hygromycin-resistant seedlings as templates, PCR detection was carried out according to the designed primers specific to the hygromycin-resistant gene.

### 4.14. Statistical Analysis

Embryogenic callus induction rate (%) = number of explants producing embryogenic callus/total number of inoculated explants. Browning rate (%) = number of browned explants/total number of inoculated explants. Processing analysis used Excel and SPSS software.

## 5. Conclusions

In this study, the lily transformation receptor system was established by the induction and regeneration of embryonic callus using ‘Siberia’ and ‘Sorbonne’ as materials. Moreover, the genetic transformation system of the embryonic callus and small scales of sterile seedlings was optimized through the construction of plant expression vectors, antibiotic screening tests, and transformation condition screening. Finally, the lily cold resistance gene and the bulblet gene were homologously transformed to obtain a lily transgenic line, which provided a certain basis for lily transgenic breeding. The specific conclusions are as follows:

(1) Induction of embryogenic callus. The stems and filaments of Oriental lilies ‘Siberia’ and ‘Sorbonne’ were used as explants to induce embryogenic callus. The results showed that ‘Siberia’ is more suitable for inducing embryogenic callus with stems, and the best medium was MS + NAA 1 mg·L^−1^ + TDZ 0.1 mg·L^−1^ + 30.0 g·L^−1^ sucrose + 7.0 g·L^−1^ agar, with an induction rate of 80.7%, while ‘Sorbonne’ is more suitable for inducing embryogenic callus with filaments, and the best medium was MS + PIC 1 mg·L^−1^ + TDZ 0.1 mg·L^−1^ + 30.0 g·L^−1^ sucrose + 7.0 g·L^−1^ agar, and the induction rate was 91.7%.
(2) Hygromycin and cephalosporin susceptibility tests. The critical concentrations of hygromycin in the embryonic callus of ‘Siberia’ and ‘Sorbonne’ are 35 mg/L and 50 mg/L, respectively. The critical concentrations of hygromycin in the small scales of sterile seedlings are 60 mg/L and 50 mg/L, respectively. Their optimum concentration of cephalosporin is 300 mg·L^−1^.
(3) Optimization of genetic transformation systems. The most suitable transformation conditions for the ‘Siberia’ embryonic callus were 0.4 OD600 and 15 min of infection time, resulting in a transformation rate of 60.0%. The optimal transformation conditions of the ‘Sorbonne’ small scales of sterile seedlings were 0.6 OD600 and 20 min infection time, resulting in a transformation rate of 60.0%. However, the ‘Sorbonne’ embryonic callus and scales of ‘Siberia’ sterile seedlings are not suitable as transformation receptors.

## Figures and Tables

**Table 1:** The effects of different media on callus induction from the stem axis of ‘Siberia’.

Medium Number	NAA Concentration (mg·L^−1^)	TDZ Concentration (mg·L^−1^)	Callus Induction Rate (%)	Browning Rate (%)
N1	1	0.1	80.7 ± 4.38 a	19.3 ± 4.38 d
N2	1	0.3	61.1 ± 3.20 b	38.9 ± 3.20 c
N3	1	0.5	27.8 ± 3.20 d	72.2 ± 3.20 a
N4	2	0.1	42.6 ± 4.91 c	57.4 ± 4.91 b
N5	2	0.3	57.4 ± 1.83 b	42.6 ± 1.83 c
N6	2	0.5	63.0 ± 4.88 b	37.0 ± 4.88 c

Note: normal letters in every column indicate significant differences at 0.05 level by Duncan’s multiple range test.

**Table 2:** The effects of different media on callus induction from the stem axis of ‘Sorbonne’.

Medium Number	NAA Concentration (mg·L^−1^)	TDZ Concentration (mg·L^−1^)	Callus Induction Rate (%)	Browning Rate (%)
N1	1	0.1	13.0 ± 1.87 a	87.0 ± 1.83 c
N2	1	0.3	5.6 ± 0.00 bc	94.4 ± 0.00 ab
N3	1	0.5	5.6 ± 3.2 bc	94.4 ± 3.20 ab
N4	2	0.1	3.7 ± 1.87 c	96.3 ± 1.87 a
N5	2	0.3	11.1 ± 0.00 bc	88.9 ± 0.00 bc
N6	2	0.5	7.4 ± 1.83 abc	92.6 ± 1.83 abc

Note: normal letters in every column indicate significant differences at 0.05 level by Duncan’s multiple range test.

**Table 3:** The effects of different media on callus induction from filaments of ‘Siberia’.

Medium Number	PIC Concentration (mg·L^−1^)	TDZ Concentration (mg·L^−1^)	Callus Induction Rate (%)	Browning Rate (%)
P1	1	0.0	38.9 ± 2.78 b	15.3 ± 1.39 d
P2	1	0.1	47.2 ± 2.78 a	23.6 ± 1.40 c
P3	1	0.2	11.1 ± 1.39 c	55.6 ± 2.78 a
P4	1	0.3	33.3 ± 0.00 b	33.3 ± 4.81 b
P5	1	0.4	33.3 ± 4.81 b	33.3 ± 0.00 b
P6	1	0.5	36.1 ± 1.39 b	13.9 ± 1.39 d

Note: normal letters in every column indicate significant differences at 0.05 level by Duncan’s multiple range test.

**Table 4:** The effects of different media on callus induction from filaments of ‘Sorbonne’.

Medium Number	PIC Concentration (mg·L^−1^)	TDZ Concentration (mg·L^−1^)	Callus Induction Rate (%)	Browning Rate (%)
P1	1	0.0	49.5 ± 2.82 d	0.00
P2	1	0.1	91.7 ± 4.81 a	0.00
P3	1	0.2	77.8 ± 2.78 bc	0.00
P4	1	0.3	83.3 ± 0.00 abc	0.00
P5	1	0.4	86.1 ± 1.39 ab	0.00
P6	1	0.5	75.0 ± 2.41 c	0.00

Note: normal letters in every column indicate significant differences at 0.05 level by Duncan’s multiple range test.

**Table 5:** The effects of different transformation conditions on embryogenic callus of ‘Siberia’.

OD 600 Infection Concentration	Infection Time (min)	Infected Embryogenic Callus Number	Resistant Embryogenic Callus Number	Embryogenic Callus Transformation Rate (%)
0.2	10	30	9	30.0%
0.2	15	30	11	36.7%
0.2	20	30	12	40.0%
0.4	10	30	14	46.7%
0.4	15	30	18	60.0%
0.4	20	30	13	43.3%

**Table 6:** The effects of different transformation conditions on sterile scales of ‘Sorbonne’.

OD 600 Infection Concentration	Infection Time (min)	Infected Sterile Seedling Small Scales Number	Induced Number of Resistant Buds	Resistant Transformation Rate (%)
0.6	10	30	7	23.3%
0.6	15	30	10	33.3%
0.6	20	30	18	60.0%
0.8	10	30	16	53.3%
0.8	15	30	13	43.3%
0.8	20	30	7	23.3%

**Table 7:** The effects of different transformation conditions on sterile scales of ‘Siberia’.

OD 600 Infection Concentration	Infection Time (min)	Infected Sterile Seedling Small Scales Number	Induced Number of Resistant Buds	Resistant Transformation Rate (%)
0.6	10	30	0	0
0.6	15	30	0	0
0.6	20	30	1	3.3
0.8	10	30	0	0
0.8	15	30	1	3.3
0.8	20	30	4	13.3

## Appendix A

**Table A1:** Media used for *Lilium* explant transformation.

Media	Composition
	the stem axis of ‘Siberia’ and ‘Sorbonne’
induction medium	MS + NAA (1, 2) mg/L + TDZ (0.1, 0.3, 0.5) mg/L + 30.0 g/L sucrose + 7.0 g/L agar
regeneration medium	MS + 30.0 g/L sucrose + 7.0 g/L agar
expansion medium	MS + NAA 1 mg/L + 90.0 g/L sucrose + 7.0 g/L agar
	the filament of ‘Siberia’ and ‘Sorbonne’
induction medium	MS + PIC 1.0 mg/L + TDZ (0.1, 0.2, 0.3, 0.4, 0.5) mg/L + 30.0 g/L sucrose + 7.0 g/L agar
regeneration medium	MS + 30.0 g/L sucrose + 7.0 g/L agar
expansion medium	MS + NAA 1 mg/L + 90.0 g/L sucrose + 7.0 g/L agar
	the sterile scales of ‘Siberia’
induction medium	MS + 6-BA 1 mg/L + NAA 0.4 mg/L + 30.0 g/L sucrose + 7.0 g/L agar
regeneration medium	MS + 30.0 g/L sucrose + 7.0 g/L agar
expansion medium	MS + NAA 1 mg/L + 90.0 g/L sucrose + 7.0 g/L agar
	the sterile scales of ‘Sorbonne’
induction medium	MS + 6-BA 0.5 mg/L + NAA 1 mg/L + 30.0 g/L sucrose + 7.0 g/L agar
regeneration medium	MS + 30.0 g/L sucrose + 7.0 g/L agar
expansion medium	MS + NAA 1 mg/L + 90.0 g/L sucrose + 7.0 g/L agar
	the embryogenic callus of ‘Siberia’
pre-culture medium	MS + NAA 1 mg/L + TDZ 0.1 mg/L + 30.0 g/L sucrose + 7.0 g/L agar
co-culture medium	MS + 1 mg/L NAA + 0.1mg/L TDZ + 100 μmol/L AS + 30.0 g/L sucrose + 7.0 g/L agar
screening medium	MS + 1 mg/L NAA + 0.1 mg/L TDZ + 30.0 g/L sucrose + 7.0 g/L agar + 35 mg/L Hyg + 300 mg/L Cef
	the embryogenic callus of ‘Sorbonne’
pre-culture medium	MS+ 1.0 mg/L PIC + 0.1 mg/L TDZ + 30.0 g/L sucrose + 7.0 g/L agar
co-culture medium	MS + 1.0 mg/L PIC + 0.1 mg/L TDZ + 100 μmol/L AS + 30.0 g/L sucrose + 7.0 g/L agar
screening medium	MS + 1.0 mg/L PIC + 0.1 mg/L TDZ + 30.0 g/L sucrose + 7.0 g/L agar + 50 mg/L Hyg + 300 mg/L Cef;
	the sterile seedling small scales of ‘Siberia’
pre-culture medium	MS + 6-BA 1mg/L + NAA 0.4 mg/L + 30.0g/L sucrose + 7.0 g/L agar
co-culture medium	MS + 1 mg/L6-BA + 0.4 mg/L NAA + 100 μmol/L AS + 30.0 g/L sucrose + 7.0 g/L agar
screening medium	MS + 1 mg/L 6-BA + 0.4 mg/L NAA + 30.0 g/L sucrose + 7.0 g/L agar + 60 mg/L Hyg + 300 mg/L Cef
	the sterile seedling small scales of ‘Sorbonne’
pre-culture medium	MS + 6-BA 0.5 mg/L + NAA 1 mg/L + 30.0 g/L sucrose + 7.0 g/L agar
co-culture medium	MS + 0.5 mg/L 6-BA + 1 mg/L NAA + 100 μmol/L AS + 30.0 g/L sucrose + 7.0 g/L agar
screening medium	MS + 0.5 mg/L 6-BA + 1 mg/LNAA + 30.0 g/L sucrose + 7.0 g/L agar 50 mg/LHyg + 300 mg/L Cef

**Table A2:** Primers used in this study.

Primer Name	Primer Sequence (5′-3′)
LaKNOX1-F	ATGGATGGCTTCACCCATCTC
LaKNOX1-R	CGGGCCCAGGCGGTATGT
LlNAC2-F	ATGGGCGGTCCAGATCTTCA
LlNAC2-R	GAACGGCTTCAGCAAGTGCA
Hyg-F	ATGGATGGCTTCACCCATCTC
Hyg-R	CGGGCCCAGGCGGTATGT

**Table A3:** Synthesize PCR products with restriction sites.

Reagent	Volume/μL
pBI121-LaKNOX1/LlNAC2-GFP	2
forward primer (10 μM)	2
Reverse primer (10 μM)	2
2× Phanta^®^ Max Master Mix (Dye Plus)	10
ddH_2_O	4
total volume	20

**Table A4:** The system of double enzyme digestion.

Reagent	Volume/μL
pCAMBIA1300-GUS	40
Xbal (15 U/μL)	2.5
SalI (10 U/μL)	2.5
Cutsmart	5
total volume	50

**Table A5:** The system of vector and target gene ligation.

Reagent	Volume/μL
pCAMBIA1300-GUS double digestion linear plasmid	>1
target gene	>1
5× CE II buffer	4
Exnase II	2
ddH_2_O	to 20
total volume	20

## References

[1] Liu Z., Zhao X., Wang W., Ke Z., Wang S. (2014). The diversity and application of Garden Lily. Mod. Landsc. Archit..

[2] Wang J.M., Ma S.L., Li W.Q., Wang Q., Cao H.Y., Gu J.H., Lu Y.M. (2016). Genetic variability and diversity of the main resources of lily assessed via phenotypic characters, pollen morphology, and ISSR markers. Genet. Mol. Res..

[3] Li W., Yong Y., Zhang Y., Lyu Y. (2019). Transcriptional regulatory network of GA floral induction pathway in LA Hybrid Lily. Int. J. Mol. Sci..

[4] Zhang Y., Yong Y.B., Wang Q., Lu Y.M. (2018). Physiological and molecular changes during lily underground stem axillary bulbils formation. Russ. J. Plant Physiol..

[5] Zambryski P., Joos H., Genetello C., Leemans J., Van Montagu M., Schell J. (1983). Ti plasmid vector for the introduction of DNA into plant cells without alteration of their normal regeneration capacity. EMBO J..

[6] Rai G.K., Rai N.P., Kumar S., Yadav A., Rathaur S., Singh M. (2012). Effects of explant age, germination medium, pre-culture parameters, inoculation medium, pH, washing medium, and selection regime on Agrobacterium-mediated transformation of tomato. In Vitro Cell. Dev. Biol.-Plant.

[7] Song S., Yan R., Wang C., Wang J., Sun H. (2020). Improvement of a genetic transformation system and preliminary study on the function of *LpABCB21* and *LpPILS7* based on somatic embryogenesis in *Lilium pumilum* DC. Fisch. Int. J. Mol. Sci..

[8] Cohen A., Meredith C.P. (1992). Agrobactierium-mediated transformation of *Lilium*. Acta Hortic..

[9] Dong W., Mao Y.F., Li W. (2007). Factors influencing T-DNA transferring into pollen of lily in vitro. Russ. J. Plant Physiol..

[10] Ogaki M., Furuichi Y., Kuroda K., Chin D.P., Ogawa Y., Mii M. (2008). Importance of co-cultivation medium pH for successful Agrobacterium-mediated transformation of *Lilium x formolongi*. Plant Cell Rep..

[11] Liu J., Zhang J., Xu B., Jia C., Zhang J., Tan G., Jin Z. (2011). Regeneration and production of transgenic *Lilium longiflorum* via *Agrobacterium tumefaciens*. In Vitro Cell. Dev. Biol.-Plant.

[12] Yong Y., Zhang Y., Lyu Y. (2021). Functional characterization of *Lilium lancifolium* cold-responsive *Zinc Finger Homeodomain* (*ZFHD*) gene in abscisic acid and osmotic stress tolerance. PeerJ.

[13] Yuan G., Wu Z., Liu X., Li T., Teng N. (2021). Characterization and functional analysis of *LoUDT1*, a *bHLH* transcription factor related to anther development in the lily oriental hybrid Siberia (*Lilium* spp.). Plant Physiol. Biochem..

[14] Liu B., Zhou Y., Lan W., Zhou Q., Li F., Chen F., Bao M., Liu G. (2019). *LlDREB1G*, a novel *DREB* subfamily gene from *Lilium longiflorum*, can enhance transgenic Arabidopsis tolerance to multiple abiotic stresses. Plant Cell Tissue Organ Cult. (PCTOC).

[15] Li J., Chai M., Zhu X., Zhang X., Li H., Wang D., Xing Q., Zhang J., Sun M., Shi L. (2019). Cloning and expression analysis of *LoCCD8* during IAA-induced bulbils outgrowth in lily (Oriental Hybrid ‘Sorbonne’). J. Plant Physiol..

[16] Zhang Q., Zhao Y.Q., Gao X., Jia G.X. (2021). Analysis of miRNA-mediated regulation of flowering induction in *Lilium × formolongi*. BMC Plant Biol..

[17] Cao L., Liu D., Jiang F., Wang B., Wu Y., Che D., Fan J. (2022). Heterologous Expression of *LiSEP3* from Oriental Lilium Hybrid ‘Sorbonne’ Promotes the Flowering of *Arabidopsis thaliana* L.. Mol. Biotechnol..

[18] Kurokawa K., Kobayashi J., Nemoto K., Nozawa A., Sawasaki T., Nakatsuka T. (2020). Expression of *LhFT1*, the Flowering Inducer of Asiatic Hybrid Lily, in the Bulb Scales. Front. Plant Sci..

[19] Yang J., Meng J., Liu X., Hu J., Zhu Y., Zhao Y., Jia G., He H., Yuan T. (2021). Integrated mRNA and small RNA sequencing reveals a regulatory network associated with flower color in oriental hybrid lily. Plant Physiol. Biochem..

[20] Cao Y., Xu L., Xu H., Yang P., He G., Tang Y., Qi X., Song M., Ming J. (2021). *LhGST* is an anthocyanin-related glutathione S-transferase gene in Asiatic hybrid lilies (*Lilium* spp.). Plant Cell Rep..

[21] Zhang T., Guo Y., Shi X., Yang Y., Chen J., Zhang Q., Sun M. (2020). Overexpression of *LiTPS2* from a cultivar of lily (*Lilium* ‘Siberia’) enhances the monoterpenoids content in tobacco flowers. Plant Physiol. Biochem..

[22] Abbas F., Ke Y., Zhou Y., Ashraf U., Li X., Yu Y., Yue Y., Ahmad K.W., Yu R., Fan Y. (2020). Molecular cloning, characterization and expression analysis of *LoTPS2* and *LoTPS4* involved in floral scent formation in oriental hybrid Lilium variety ‘Siberia’. Phytochemistry.

[23] Yan R., Wang Z., Ren Y., Li H., Liu N., Sun H. (2019). Establishment of efficient genetic transformation systems and application of CRISPR/Cas9 genome editing technology in *Lilium pumilum* DC. Fisch. And *Lilium longiflorum* White Heaven. Int. J. Mol. Sci..

[24] Bakhshaie M., Khosravi S., Azadi P., Bagheri H., van Tuyl J.M. (2016). Biotechnological advances in *Lilium*. Plant Cell Rep..

[25] Roiloa S.R., Retuerto R. (2006). Small-scale Heterogeneity in Soil Quality Influences Photosynthetic Efficiency and Habitat Selection in a Clonal Plant. Ann. Bot..

[26] Wang J., Yang Y., Liu X., Huang J., Wang Q., Gu J., Lu Y. (2014). Transcriptome profiling of the cold response and signaling pathways in *Lilium lancifolium*. BMC Genom..

[27] Wang J., Wang Q., Yang Y., Liu X., Gu J., Li W., Ma S., Lu Y. (2014). De novo assembly and characterization of stress transcriptome and regulatory networks under temperature, salt and hormone stresses in *Lilium lancifolium*. Mol. Biol. Rep..

[28] Kerstetter R., Vollbrecht E., Lowe B., Veit B., Yamaguchi J., Hake S. (1994). Sequence analysis and expression patterns divide the maize *knotted1-like* homeobox genes into two classes. Plant Cell.

[29] Reiser L., Sánchez-Baracaldo P., Hake S. (2000). Knots in the family tree: Evolutionary relationships and functions of *knox* homeobox genes. Plant Mol. Biol..

[30] Sinha N.R., Williams R.E., Hake S. (1993). Overexpression of the maize homeo box gene, *KNOTTED-1*, causes a switch from determinate to indeterminate cell fates. Genes Dev..

[31] Clark S.E., Jacobsen S.E., Levin J.Z., Meyerowitz E.M. (1996). The clavata and shoot meristemless loci competitively regulate meristem activity in Arabidopsis. Development.

[32] Kerstetter R.A., Laudencia-Chingcuanco D., Smith L.G., Hake S. (1997). Loss-of-function mutations in the maize homeobox gene, *knotted1*, are defective in shoot meristem maintenance. Development.

[33] Chan R.L., Gago G.M., Palena C.M., Gonzalez D.H. (1998). Homeoboxes in plant development. Biochim. Biophys. Acta.

[34] Smith L.G., Greene B., Veit B., Hake S. (1992). A dominant mutation in the maize homeobox gene, *Knotted-1*, causes its ectopic expression in leaf cells with altered fates. Development.

[35] Hay A., Tsiantis M. (2010). *KNOX* genes: Versatile regulators of plant development and diversity. Development.

[36] Hake S., Smith H.M., Holtan H., Magnani E., Mele G., Ramirez J. (2004). The role of *knox* genes in plant development. Annu. Rev. Cell Dev. Biol..

[37] Frugis G., Giannino D., Mele G., Nicolodi C., Chiappetta A., Bitonti M.B., Innocenti A.M., Dewitte W., Van Onckelen H., Mariotti D. (2001). Overexpression of *KNAT1* in lettuce shifts leaf determinate growth to a shoot-like indeterminate growth associated with an accumulation of isopentenyl-type cytokinins. Plant Physiol..

[38] Kim D., Cho Y.H., Ryu H., Kim Y., Kim T.H., Hwang I. (2013). *BLH1 and KNAT3* modulate ABA responses during germination and early seedling development in Arabidopsis. Plant J..

[39] Zhong R., Lee C., Zhou J., McCarthy R.L., Ye Z.H. (2008). A battery of transcription factors Involved in the regulation of secondary cell wall biosynthesis in *Arabidopsis*. Plant Cell..

[40] Truernit E., Haseloff J. (2007). A Role for *KNAT* Class II Genes in Root Development. Plant Signal Behav..

[41] Bhargava A., Mansfield S.D., Hall H.C., Douglas C.J., Ellis B.E., Bhargava A., Mansfield S.D., Hall H.C. (2010). MYB75 Functions in Regulation of Secondary Cell Wall Formation in the Arabidopsis Inflorescence Stem. Plant Physiol..

[42] Li E., Wang S., Liu Y., Chen J.G., Douglas C.J. (2011). *OVATE FAMILY PROTEIN4* (*OFP4*) interaction with *KNAT7* regulates secondary cell wall formation in *Arabidopsis thaliana*. Plant J..

[43] Li E., Bhargava A., Qiang W., Friedmann M.C., Forneris N., Savidge R.A., Johnson L.A., Mansfield S.D., Ellis B.E., Douglas C.J. (2012). The Class II *KNOX* gene *KNAT7* negatively regulates secondary wall formation in *Arabidopsis* and is functionally conserved in *Populus*. New Phytol..

[44] Scofield S., Murray J.A. (2006). *KNOX* Gene Function in Plant Stem Cell Niches. Plant Mol. Biol..

[45] Huang Z., Meilan R., Woeste K. (2009). A *KNAT3-like* homeobox gene from *Juglans nigra* L. *JnKNAT3-like*, highly expressed during heartwood formation. Plant Cell Rep..

[46] Moreno-Pachón N.M. (2017). Mechanisms of Vegetative Propagation in Bulbs: A Molecular Approach. Ph.D. Thesis.

[47] Chung P.J., Jung H., Choi Y.D., Kim J.K. (2018). Genome-wide analyses of direct target genes of four rice *NAC*-domain transcription factors involved in drought tolerance. BMC Genom..

[48] Wu Y., Deng Z., Lai J., Zhang Y., Yang C., Yin B., Zhao Q., Zhang L., Li Y., Yang C. (2009). Dual function of *Arabidopsis ATAF1* in abiotic and biotic stress responses. Cell Res..

[49] Yong Y., Zhang Y., Lyu Y. (2019). A Stress-Responsive *NAC* Transcription Factor from *Tiger Lily* (*LlNAC2*) Interacts with *LlDREB1* and *LlZHFD4* and Enhances Various Abiotic Stress Tolerance in *Arabidopsis*. Int. J. Mol. Sci..

[50] Cao H., Wang L., Nawaz M.A., Niu M., Sun J., Xie J., Kong Q., Huang Y., Cheng F., Bie Z. (2017). Ectopic expression of Pumpkin *NAC* transcription factor *CmNAC1* improves multiple abiotic stress tolerance in *Arabidopsis*. Front. Plant Sci..

[51] Wu D., Sun Y., Wang H., Shi H., Su M., Shan H., Li T., Li Q. (2018). The *SlNAC8* gene of the halophyte Suaeda liaotungensis enhances drought and salt stress tolerance in transgenic *Arabidopsis thaliana*. Gene.

[52] Wang L., Li Z., Lu M., Wang Y. (2017). *ThNAC13*, a *NAC* transcription factor from *Tamarix hispida*, confers salt and osmotic stress tolerance to transgenic *Tamarix* and *Arabidopsis*. Front. Plant Sci..

[53] Lu X., Zhang X., Duan H., Lian C., Liu C., Yin W., Xia X. (2018). Three stress-responsive *NAC* transcription factors from *Populus euphratica* differentially regulate salt and drought tolerance in transgenic plants. Physiol. Plant..

[54] Sun D.Y., Niu L.X., Zhang Y.L., Zhang X.G., Li S.H. (2016). Study on callus induction and rooting of wild lily organs. Res. Prog. Ornam. Hortic. China.

[55] Liu X., Gu J., Wang J., Lu Y. (2014). Lily breeding by using molecular tools and transformation systems. Mol. Biol. Rep..

[56] Qi Y., Du L., Quan Y., Tian F., Liu Y., Wang Y. (2014). Agrobacterium-mediated transformation of embryogenic cell suspension cultures and plant regeneration in *Lilium tenuifolium* Oriental × trumpet ‘Robina’. Acta Physiol. Plant..

[57] Mori S., Adachi Y., Horimoto S., Suzuki S., Nakano M. (2005). Callus formation and plant regeneration in various *Lilium* species and cultivars. In Vitro Cell. Dev. Biol. Plant.

[58] Zhang J., Gai M., Li X., Li T., Sun H. (2016). Somatic embryogenesis and direct as well as indirect organogenesis in *Lilium pumilum* DC. Fisch., an endangered ornamental and medicinal plant. Biosci. Biotechnol. Biochem..

[59] Loyola-Vargas V.M., Ochoa-Alejo N. (2016). The Many Ways of Somatic Embryo Initiation.

[60] Gnanaraj M., Sneka C., Sisubalan N., Baburajan R., Manikandan R., Muneeswaran T. (2022). Polyethylene glycol induced somatic embryogenesis and plant regeneration from cotyledons of *Vigna radiata* (L.) Wilczek. South Afr. J. Bot..

[61] Nakano M., Otani M. (2020). Plant regeneration and Agrobacterium-mediated genetic transformation systems in *liliaceous* ornamental plants. Plant Biotechnol..

[62] Suzuki S., Oota M., Nakano M. (2002). Embryogenic callus induction from leaf explants of the *Liliaceous* ornamental plant, *Agapanthus praecox* ssp. *orientalis* (Leighton) *Leighton*-Histological study and response to selective agents. Sci. Hortic..

[63] Ahmed R.I., Ding A., Xie M., Kong Y. (2018). Progress in Optimization of Agrobacterium-Mediated Transformation in Sorghum (*Sorghum bicolor*). Int. J. Mol. Sci..

[64] Pushyami B., Beena M.R., Sinha M.K., Kirti P.B. (2011). In vitro regeneration and optimization of conditions for Agrobacterium mediated transformation in jute, *Corchorus capsularis*. J. Plant Biochem. Biotechnol..

[65] Li S., Yang N., Chen L. (2022). Paraffin section observation of flower bud differentiation of Chimonanthus praecox in Kunming and comparison of the differentiation processes in different regions, China. Hortic. Plant J..

[66] Zhou N.F., Zhang J.P., Liu H., Weiwei Z., Pei D. (2018). New protocols for paraffin sections of heterogeneous tissues of woody plants. Chin. Bull. Bot..

[67] Zhang X.W., Liu L.P. (2022). Research on dandelion paraffin sectioning technology. Spec. Econ. Anim. Plants.

